# Prevalence of hearing impairment and associated factors in school-aged children and adolescents: a systematic review^☆^

**DOI:** 10.1016/j.bjorl.2018.10.009

**Published:** 2018-12-01

**Authors:** Aryelly Dayane da Silva Nunes, Carla Rodrigues de Lima Silva, Sheila Andreoli Balen, Dyego Leandro Bezerra de Souza, Isabelle Ribeiro Barbosa

**Affiliations:** a Universidade Federal do Rio Grande do Norte (UFRN), Programa de Pós-Graduação em Saúde Coletiva, Natal, RN, Brazil; b Universidade Federal do Rio Grande do Norte (UFRN), Departamento de Fonoaudiologia, Natal, RN, Brazil; c Universidade Federal do Rio Grande do Norte (UFRN), Departamento de Saúde Coletiva, Natal, RN, Brazil; d Universidade Federal do Rio Grande do Norte (UFRN), Faculdade de Ciências da Saúde de Trairi (FACISA), Santa Cruz, RN, Brazil

**Keywords:** Hearing loss, Child, Adolescent, Prevalence, Epidemiologic factors, Perda auditiva, Criança, Adolescente, Prevalência, Fatores epidemiológicos

## Abstract

**Introduction:**

Hearing impairment is one of the communication disorders of the 21st century, constituting a public health issue as it affects communication, academic success, and life quality of students. Most cases of hearing loss before 15 years of age are avoidable, and early detection can help prevent academic delays and minimize other consequences.

**Objective:**

This study researched scientific literature for the prevalence of hearing impairment in school-aged children and adolescents, with its associated factors. This was accomplished by asking the defining question: “What is the prevalence of hearing impairment and its associated factors in school-aged children and adolescents?”

**Methods:**

Research included the databases PubMed/MEDLINE, LILACS, Web of Science, Scopus and SciELO, and was carried out by two researchers, independently. The selected papers were analyzed on the basis of the checklist provided by the report Strengthening the Reporting of Observational Studies in Epidemiology.

**Results:**

From the 463 papers analyzed, 26 fulfilled the criteria and were included in the review presented herein. The detection methods, as well as prevalence and associated factors, varied across studies. The prevalence reported by the studies varied between 0.88% and 46.70%. Otologic and non-otologic factors were associated with hearing impairment, such as middle ear and air passage infections, neo- and post-natal icterus, accumulation of cerumen, family history, suspicion of parents, use of earphones, age and income.

**Conclusion:**

There is heterogeneity regarding methodology, normality criteria, and prevalence and risk factors of studies about hearing loss in adolescents and school-aged children. Nevertheless, the relevance of the subject and the necessity of early interventions are unanimous across studies.

## Resumo

**Introdução:**

A deficiência auditiva é um dos distúrbios de comunicação do século XXI, constitui um problema de saúde pública, pois afeta a comunicação, o sucesso acadêmico e a qualidade de vida dos estudantes. A maioria dos casos de perda auditiva antes dos 15 anos é evitável e a detecção precoce pode ajudar a evitar atrasos acadêmicos e minimizar outras consequências.

**Objetivo:**

Este estudo investigou a literatura científica sobre a prevalência da deficiência auditiva em crianças e adolescentes em idade escolar, com seus fatores associados. Isso foi feito através da questão norteadora: “Qual a prevalência da deficiência auditiva e seus fatores associados em crianças e adolescentes em idade escolar?”

**Método:**

A pesquisa compreendeu as bases de dados PubMed/MEDLINE, LILACS, Web of Science, Scopus e SciELO e foi feita de forma independente por dois pesquisadores. Os artigos selecionados foram analisados com base na lista de verificação fornecida pelo relatório Strengthening the Reporting of Observational Studies in Epidemiology.

**Resultados:**

Dos 463 artigos analisados, 26 preencheram os critérios e foram incluídos na revisão aqui apresentada. Os métodos de detecção, assim como a prevalência e os fatores associados, variaram entre os estudos. A prevalência relatada pelos estudos variou entre 0,88% e 46,70%. Fatores otológicos e não otológicos foram associados à deficiência auditiva, como infecções da orelha média e das vias aéreas, icterícia neonatal e pós-natal, acúmulo de cerúmen, histórico familiar, suspeita dos pais, uso de fones de ouvido, idade e renda.

**Conclusão:**

Há heterogeneidade quanto à metodologia, aos critérios de normalidade e, consequentemente, à prevalência e aos fatores associados nos estudos sobre da perda auditiva em adolescentes e crianças em idade escolar. No entanto, a relevância do assunto e a necessidade de intervenções precoces são unânimes entre os estudos.

## Introduction

In the 21st century, communication disorders (which include hearing impairment, HI) constitute a serious concern within public health; if not treated, there are negative effects on the economic well-being of a society in the era of communication.^1^ The problem deserves to be highlighted, as the sense of hearing is essential for the development of speech, language and learning,^2^ and the higher the degree of hearing impairment, the greater the difficulties in perceiving and distinguishing speech, including language deficits.^3^

In children under the age of 15, 60% of hearing loss occur due to avoidable causes,^4^ and estimates indicate that 1.1 billion people around the world could be at risk for hearing impairment due to unsafe hearing practices, such as the use of individual audio devices.^5^ Adolescents deserve close attention, as they are exposed to high levels of non-occupational noise.^5^, ^6^ Some factors associated with hearing impairment include infections of the superior air passages^7^ and middle ear,^8^, ^9^, ^10^ in addition to the presence of cerumen obstructing the external acoustic meatus,^9^, ^10^, ^11^ as these can interfere in the transmission of the hearing stimulus. However, despite the fact that the causes of HI can be identified in children and adolescents, data are limited regarding possible risk factors for acquired HI.^8^

Early detection of HI can help prevent academic delays,^10^ besides being a determinant for productivity and life quality of the potential bearer of HI.^12^ Auditory tests are indicated for the early detection of hearing disorders.^7^ Therefore the need or deeper knowledge on the prevalence and associated factors for hearing impairment in school-aged individuals is evident. Prevention and intervention actions could then be carried out to minimize the negative consequences of HI in the life of individuals. The objective of this study is to carry out a systematic review in the scientific literature on the prevalence of hearing impairment and its associated factors in school-aged individuals.

## Methods

A systematic literature review was carried out, guided by the question: “What is the prevalence of hearing impairment and its associated factors in school-aged children and adolescents?” The databases consulted were PubMed/MEDLINE, LILACS, Web of Science, Scopus and SciELO. The main descriptors related to the investigated subject were crossed: “prevalence”, “epidemiology”, “cross-sectional studies “hearing”, “hearing loss”, “hearing disorders”, “school health services”, “school health”, “child”, and “adolescent”, as shown by the strategies depicted in Table 1.

**Table 1:** Search strategy for the selected databases.

Pubmed	((((prevalence and epidemiology)) AND cross-sectional studies) AND (hearing loss or hearing)) AND (child or adolescent) (school health services or school health)
Web of science	(TS = (prevalence) AND TS = (Hearing loss or hearing) AND TS = (cross-sectional studies) AND TS = (child or adolescent))
Scopus	ALL(prevalence) AND ALL(“cross-sectional studies”) AND ALL(“hearing loss” OR “hearing disorders”) AND ALL(“school health services” OR “school health”) AND ALL(child OR adolescent)
Lilacs	“Pérdida Auditiva” OR “hearing loss” OR “perda auditiva” [Words] and Prevalência OR Prevalencia OR Prevalence [Words] and Criança OR Niño OR child [Words]
Scielo	((prevalence AND (“hearing loss” OR hearing))) AND (child OR adolescent)

The review included only the studies that were cross-sectional and presented the prevalence of hearing impairment in children and/or adolescents. Other types of studies or formats were excluded as well as cross-sectional studies that included children and/or adolescents but did not present a specific prevalence for this population. Bibliographic data compilation occurred on April 10, 2018, based on the aforementioned inclusion criteria. The first phase of the selection of papers was the exclusion of duplicate studies, followed by the reading and analysis of titles and abstracts of all identified papers. The next step was the complete reading of the selected studies, which led to the exclusion of papers that were not aligned with the review proposal. The bibliographies of the papers identified were analyzed to identify possible additional studies that could be added to the review presented herein.

The selected papers underwent methodological assessment in accordance with the checklist provided by Strengthening the Reporting of Observational Studies in Epidemiology (STROBE)^13^ for cross-sectional studies, receiving the value 1 when the item was contemplated, 0 when not contemplated and 0.5 when partially contemplated. All phases were carried out by the two first authors/researchers, independently. The study presented herein only included the papers that reached at least 60% of the score determined by the STROBE checklist, with a cutoff point established to ensure good methodological quality. Papers that did not meet the cutoff threshold were excluded. All procedures of the review presented herein were conducted in accordance with the checklist of the Reporting Items for Systematic Reviews and Meta-Analyses (PRISMA).

## Results

**Figure 1:**
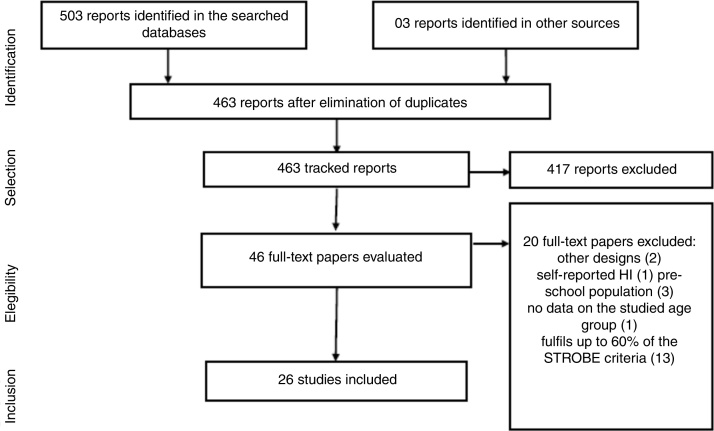
Flowchart of paper selection.

A total of 463 papers were identified, which approached the prevalence of hearing impairment in school-aged children and/or adolescents. After all the methodological steps, 26 papers were included (Fig. 1), with a description of the methodological quality shown in Table 2. The papers investigated different populations, age groups, hearing impairment diagnosis criteria and methods, revealing heterogeneity in the results.

**Table 2:** Methodological quality of the studies included, in accordance with the STROBE checklist.

Reference	TA	SJ	O	SD	S	P	V	DM	B	SS	QV	SM	P	DD	O	MR	OA	MR	L	I	G	F	Total
Al-Rowaily et al. (2012)	1	0.5	1	1	1	1	1	1	0	1	1	0.5	1	0.5	1	1	0	1	1	0.5	1	0	17
Al-Khabori et al. (2004)	1	1	1	1	0.5	1	0.5	0.5	0	1	0	0	1	1	1	1	0	1	0.5	0.5	0	1	14.5
Balen et al. (2009)	1	1	1	0.5	1	1	1	0.5	0	1	1	0.5	1	1	1	0.5	0	1	0	0.5	0	1	15.5
Baraky et al. (2012)	1	1	1	1	1	1	1	1	0.5	1	1	1	1	1	1	1	0	1	0	1	1	1	19.5
Béria et al. (2007)	1	1	1	1	1	1	1	1	0	1	1	1	1	1	1	1	1	1	1	1	0.5	1	20.5
Bevilacqua et al. (2013)	1	0.5	1	1	1	1	1	1	0	1	0.5	0	1	1	1	0.5	0	1	0	0.5	0.5	1	15.5
Chen et al. (2011)	0.5	0.5	1	1	1	0.5	1	1	0	0.5	1	1	1	1	0.5	0.5	0	1	1	1	1	1	17
Czechowicz et al. (2010)	1	1	1	1	0.5	1	1	1	0	0.5	1	1	1	1	1	1	0	1	0	1	0.5	1	17.5
Feder et al. (2017)	1	0.5	1	0.5	0.5	1	1	1	0	1	1	0.5	0.5	1	0.5	0.5	0	1	1	1	1	1	16.5
Gierek et al. (2009)	0.5	1	1	0	1	1	1	1	0	0	1	1	0.5	1	1	1	1	1	0	0.5	0.5	1	16
Gondim et al. (2012)	1	1	1	1	1	1	1	1	0.5	1	1	0.5	1	1	1	1	0	1	0	0.5	0	0	16.5
Govender et al. (2015)	1	1	1	1	1	1	0.5	1	0	1	0.5	1	0.5	1	1	1	0	1	0	1	0.5	0	16
Hong et al. (2016)	1	1	1	1	1	1	1	1	0	0.5	1	1	1	1	1	1	0	1	1	1	0.5	0	18
Jun et al. (2015)	1	1	1	1	1	0.5	1	1	0	0.5	0.5	1	1	1	1	1	0	1	1	1	1	1	18.5
Kam et al. (2013)	1	1	1	1	0.5	0.5	0.5	1	0	0	0	1	1	1	1	1	0	1	1	0	0	1	14.5
le Clercq et al. (2017)	1	1	1	0.5	1	1	1	1	0	1	1	1	1	1	1	1	0	1	1	1	1	1	19.5
Niskar et al. (1998)	0.5	1	0.5	1	0.5	1	0.5	1	0	0.5	1	1	1	1	1	1	0	1	1	1	1	1	17
Ramma et al. (2016)	1	1	1	1	1	1	1	1	1	1	1	1	1	1	1	1	1	1	1	1	1	0	21
Samelli et al. (2011)	0.5	1	1	0.5	0.5	0.5	1	1	0	0	1	1	1	1	1	1	1	1	1	1	1	1	18
Serra et al. (2014)	0.5	1	1	0	0.5	0.5	1	0.5	0	0	0.5	1	1	1	1	1	0	1	0.5	1	0.5	1	14.5
Shargorodsky et al. (2010)	1	1	1	1	1	0.5	1	1	0	0	1	1	1	1	1	1	0	1	1	1	0.5	1	17
Skarzyński et al. (2016)	0.5	1	1	0	0	0.5	0.5	1	0	0	0.5	1	1	1	0.5	1	0	1	0	1	1	1	13.5
Taha et al. (2010)	0.5	1	0	0	1	0.5	1	1	0	0	1	1	1	1	1	1	0	1	0.5	1	1	0	14.5
Tarafder et al. (2015)	1	1	1	1	0.5	1	1	1	0	1	1	1	1	1	1	1	0	1	1	0.5	0.5	1	18.5
Wake et al. (2006)	1	1	1	1	1	1	1	1	0	1	0.5	1	1	1	1	1	0	1	1	1	1	1	19.5
Westerberg et al. (2005)	1	1	1	1	0.5	1	1	1	0	1	0.5	0	1	1	1	1	0	1	1	1	0.5	1	17.5

TA, title and abstract; SJ, setting/motivation; O, objectives; SD, study design; S, settings; P, participants; V, variables; DM, data source/measurement; B, bias; SS, size of sample; QV, quantitative variables; SM, statistic methods; P, participants; DD, descriptive data; O, outcome; MR, main results; OA, other analyses; MR, main results; L, limitations; I, interpretation; G, generalization; F, funding.

The studies evaluated different age groups, and eight papers included age groups beyond children and adolescents.^11^, ^12^, ^14^, ^15^, ^16^, ^17^, ^18^, ^19^ There was variation in the diagnostic methods and normality criteria across the selected studies. Some studies utilized the auditory threshold as screening procedure,^9^, ^11^, ^12^, ^15^, ^16^, ^18^, ^19^, ^20^, ^21^, ^22^, ^23^, ^24^, ^25^, ^26^, ^27^, ^28^ automated auditory threshold,^8^, ^17^, ^29^, ^30^ audiometric screening,^14^, ^31^ and audiometric diagnosis at some point.^10^, ^32^, ^33^ Regarding normality criteria, there were differences even among those that utilized the same technique, either auditory threshold or scanning, and some studies presented a set of procedures to indicate test normality. Due to these differences, there was variation in the prevalence values encountered. Most studies did not provide the respective confidence intervals (CI) (Table 3), and some studies analyzed prevalence through different criteria and/or assessed a wider age group that what was included herein, presenting CI for some criteria.

**Table 3:** Characteristics of the included studies, with methodological quality evaluated in accordance with the STROBE checklist criteria.

Reference	City/country	Sample/population	Diagnosis method	Normality criterion	Prevalence of HI	Factors associated with HI
Al-Rowaily et al. (2012)	King Abdulaziz Medical City, Saudi Arabia	2574 (4–8 years)	Auditory threshold 1, 2 and 4 kHz^a^	20 dB	1.75% (1.25–2.25)	otitis media, cerumen, chronic otitis media, sensorineural hearing loss, tympanic perforation^b^
Al-Khabori et al. (2004)	Oman	11,400 individuals^c^	Screening at 1, 2 and 4 kHz	>25 dB Immediate re-test at 35 dB	0–9 years, 16.7% (12.71–20.76)	Cerumen, presbycusis, infections^b^, ^d^
					10–19 years, 33.3% (27.63–38.91)	
Balen et al. (2009)	Itajaí, Brazil	419 (0–14 years)	4–14 years: Auditory threshold at 1, 2 and 4 kHz, acoustic reflexes and tympanometry	>15 dB for best ear	16.84%	Associated factors not included in the study.
Baraky et al. (2012)	Juiz de Fora, Brazil	267 (4–19 years)	Otoscopy Auditory threshold at 1, 2 and 4 kHz Questionnaire	Incapacitating hearing loss (WHO)	3.03% (8–267)	Buzz, >60 years, low education level^d^
Béria et al. (2007)	Canoas, Brazil	776 (4–19 years)	Auditory threshold at 1, 2 and 4 kHz	Incapacitating hearing loss (WHO)	4–9 years: 12%; 10–19 years: 7.1%	Income and education level^d^
					Incapacitating: 4–9 years: 5.3%; 10–19 years: 2.2%	
Bevilacqua et al. (2013)	Monte Negro, Brazil	577 individuals^c^	Otoscopy Auditory threshold at 1, 2 at 4 kHz	0–29 dB no compromise; 30–40 dB slight; 41–60 dB moderate; 61–80 dB severe; >80 dB profound	3.8% (2.17–5.45) incapacitating	Associated factors not included in study.
Chen et al. (2011)	Xi’na, China	1567 (12–19 years)	Otoscopy Auditory threshold 0.25 kHz to 8 kHz Tympanometry	Auditory threshold (500–4000 Hz) > 25 dB	3.32% ear disease (30–1567)	Gender, use of portable audio devices, ototoxic drugs, HI Family history
Czechowicz et al. (2010)	Lima district, Peru	355 (6–19 years)	Pneumatic otoscopy Auditory threshold 0.25, 0.5, 1, 2, 4, 8 kHz Tympanometry Academic performance and questionnaire applied with responsible adult	>25 dB	6.9% (4.2%–9.6%)	Income, poverty. Neonatal icterus, hospitalization, recurrent middle ear infections, HI Family history <35 years, tympanic membrane abnormality, impacted cerumen, tube dysfunction
Feder et al. (2017)	Canada	1879 (6–19 years)	Auditory threshold at 0.5 kHz to 8 kHz	>20 dB	4.7%	Associated factors not included in study.
			EOAPD	>26 dB and “passing” in three out of four test frequencies (2, 3, 4 and 5 kHz) with SR 6 dB		
Gierek et al. (2009)	Upper Silesia, Poland	8885 (6–14 years)	Screening at 1, 2 and 4 kHz Speech in noise Test with figures and test with words^a^	25 dB NA	10.3% failed	Dysfunction of auditory tubes due to upper airway infection
				90% correct; 75% correct	6% confirmed HI	
Gondim et al. (2012)	Itajaí, Brazil	35 (4–9 years)	Questionnaire Otoscopy Auditory threshold at 1, 2 and 4 kHz Tympanometry Acoustic reflexes	Incapacitating hearing loss (WHO)	2.86%	Presbycusis, idiopathy, cerumen, chronic otitis media, otosclerosis, noise induced hearing loss, labyrinthopathy.^b^, ^d^
Govender et al. (2015)	Durban, South Africa	241 (1st year students)	Otoscopy Tympanometry Auditory threshold at 0.5, 1, 2 and 4 kHz	20 dB NA	24%	The studied factors did not present statistical significance
Hong et al. (2016)	Korea	1534 (13–18 years)	Automated auditory threshold at 0.5 kHz to 6 kHz	>25 dB 0.5, 1, 2 and 3 kHz	2.2% (1.3–3.7) unilateral	Age, tympanometry, income, use of earphones with thresholds >20 dB in high frequencies
					0.4% (0.2–0.9) bilateral	
Jun et al. (2015)	South Korea	2033 (12–19 years)	Automated auditory threshold 0.5 to 6 kHz	HI speech frequency: thresholds at 0.5, 1, 2, 3, 4 kHz ≥ 25 dBNA	Unilateral: 2.18% (±0.48)	Age, sex
					Bilateral: 0.34% (±0.13)	
				HI high frequency: thresholds at 3, 4, 6 kHz ≥ 25 dBNA	Unilateral: 2.81% (±0.55)	
					Bilateral: 0.83% (±0.25)	
Kam et al. (2013)	Shenzhen, China	325 (6–10 years)	Automated auditory threshold at 1, 2 and 4 kHz	>25 dB	4.92%	Associated factors not included in study.
le Clercq et al. (2017)	Rotterdam, Netherland	5368 (9–11 years)	Auditory threshold at 0.5 kHz to 8 kHz Tympanometry	>15 dB	17.50%	OM and low maternal education levels
Niskar et al. (1998)	EUA	6166 (6–19 years)	Auditory threshold at 0.5 kHz to 8 kHz	>15 dB	14.9%	Cold, sinusitis, earache, ventilation tube, self-reported on the evaluation day
Ramma et al. (2016)	Cape Town, South Africa	1000 (4–19 years)	Auditory threshold at 0.25 kHz to 8 kHz	>25 dB	4–9 (4.3%); 10–19 (2.6)	Male sex, age, hypertension, history of cranioencephalic trauma, and HI family history.^b^
Samelli et al. (2011)	Butantã, Brazil	214 (2–10 years)	Auditory assessment^a^	>15 dB, tympanogram, presence of acoustic reflexes	46.7%	Associated factors not included in the study.
Serra et al. (2014)	Córdoba, Argentina	172 (14–15 years)	Auditory threshold 0.25–8 kHz; 8–16 kHz TOAE	18 dB; reproductivity: >70% SNR; >6 dB in 3 frequencies	34.88%	Associated factors not included in study.
Shargorodsky et al. (2010)	USA	Cycle 1988–1994: 1771 (12–19 years)	Automated hearing threshold at 0.5–8 kHz. Noise-induced threshold shift	Worst ear: discrete between 15 and 25 dB NA, slight or higher >25 dB NA	Cycle 1988–1994: 14.9% (13.0–16.9)	Race/Ethnicity Poverty rate/income 3+ middle ear infections
		Cycle 2005–2006: 2288 (12–19 years)			Cycle 2005–2006: 19.5% (15.2–23.8)	
Skarzyński et al. (2016)	Tajikistan, Poland	143 (7–8 years)	Auditory threshold, questionnaires (parents and children)	25 dB	23.7%	Associated factors not included in study.
Taha et al. (2010)	Shebin El-Kom District, Egypt	555 (6–12 years)	Audiometric screening, questionnaire^a^	20 dB	20.9%	Suspicion of parents, otitis media, consumption of tobacco at home, low socio-economic level, and post-natal icterus.
Tarafder et al. (2015)	Bangladesh	899 (5–14 years)	Auditory threshold 0.5, 1, 2, 4 kHz; EOAT	30 dB	13%	Age, socioeconomic deprivation, family history, impacted ear wax, chronic suppurative otitis media, otitis media with effusion, and external otitis
Wake et al. (2006)	Melbourne, Australia	6581 (=∼7–12 years)	Auditory threshold 0.5, 1 and 2 kHz or 3, 4 and 6 kHz	>40 dB best ear	0.88% (0.66–1.15)	Poorer short term phonological memory
Westerberg et al. (2005)	Manicaland, Zimbabwe	5528 (4–20 years)	Auditory screening at 1, 2 and 4 kHz	>30 dB	2.4% (2.0–2.8)	Impacted cerumen, infections^b^

a This study includes diagnostic auditory assessment.

b These studies did not include analysis of associated factors, only analysis of the causes.

c These studies did not include specific age groups for children/adolescents.

d These studies did not include specific analysis of associated factors for the studied age group, only for general population.

Similarly, the study of associated factors was not homogeneous. Seven studies did not include analysis of associated factors besides prevalence of hearing impairment,^16^, ^22^, ^24^, ^26^, ^27^, ^29^, ^33^ and seven studies included analysis, but it was not specific for the age group of children and/or adolescents.^11^, ^12^, ^14^, ^15^, ^17^, ^18^, ^19^ Due to the low number of studies that evaluated associated factors, the causes established by the studies were indicated as associated factors in Table 3.

## Discussion

Twenty-six papers were selected for systematic review, but there was significant variation in the identification method for hearing impairment, normality criteria and investigated age groups, which consequently led to variability in the prevalence and its associated factors.

The lowest prevalence encountered was 0.88%^21^ and the highest was 46.7%.^33^ While some studies included diagnosis assessment,^7^, ^10^, ^32^ others considered incapacitating hearing loss.^11^, ^12^, ^15^, ^16^, ^18^ Some studies applied questionnaires,^9^, ^26^, ^32^, ^33^ but with different objectives. Questionnaires were applied with parents^9^, ^26^, ^32^ and school-aged individuals, to investigate potential causes of hearing changes^26^ and risk factors for HI^32^ such as health history,^9^ possible presence of buzzing and learning difficulties.^26^ However, one of the studies had the objective of developing a questionnaire as a low-cost tool for auditory screening.^33^

The prevalences found in the studies varied according to method, age group and normality criterion established by the authors and population under study; there was also variability in the study of risk factors associated with HI. Considering the studies that focused on evaluating children and/or adolescents, and considering the age group “children” limited to 12 years of age, it was verified that the same number of studies considered children,^21^, ^25^, ^26^, ^28^, ^29^, ^32^, ^33^ and both age groups (children and adolescents),^7^, ^9^, ^10^, ^20^, ^22^, ^27^, ^31^ with limited specific research on adolescents.^8^, ^23^, ^24^, ^30^ It must be highlighted that the age ranges within the age groups were not the same, nor were the sampling criteria for each study.

Some studies mixed preschoolers with school-aged individuals,^10^, ^12^, ^15^, ^19^, ^22^, ^31^, ^33^ and within these studies the most common causes for hearing impairment were impacted cerumen^10^, ^31^ and infections^31^ such as otitis media.^10^, ^31^ In these studies, prevalence varied between 1.75%^10^ and 46.7%.^33^ These higher values could be explained by the diagnosis criterion utilized, which besides audiometry, also considered Type A tympanogram and the presence of acoustic reflexes. Also, there were groups of children with higher prevalence of conductive alterations, such as diagnosis of conductive loss in 84.4%^10^ of the children with HI. However, the study that compared two age groups within the same population found similar prevalence: 1.3% for the age group 4–9 years old, and 1.4% for the age group 10–19 years old, from the analysis of the best ear.^16^

The normality criterion employed, the number of school-aged individuals included and/or the selected population could have caused such discrepancies, as the main causes of HI for younger individuals are conductive factors – otitis media with effusion (age group 4–8 years old),^10^ otitis media with effusion, associated with auditory tube dysfunction and adenoid dysplasia (age group 4–10 years old).^11^ The study that encountered the lowest prevalence evaluated a specific group of school-aged individuals, with the objective of establishing HI prevalence in those who underwent neonatal auditory screening. For this reason, those that did not undergo screening or those already diagnosed with HI were excluded.^23^ The studies did not present deep discussions on the etiology, possibly because the results originate from prevalence studies and not from diagnostic investigation. It is important to study not only the factors that lead to hearing impairment, but also the genetic causes.

The risk factors for HI in children and adolescents can be otologic or non-otologic.^9^ The consulted studies revealed different factors associated with HI such as suspicion of parents,^32^ poorer short term phonologic memory,^21^ use of personal electronic devices,^23^ middle ear infections,^8^, ^9^, ^10^, ^11^, ^18^, ^31^ infections such as measles, meningitis, mumps and maternal German measles,^31^ tube dysfunction,^7^, ^9^ cerumen,^9^, ^10^, ^11^, ^14^, ^18^, ^20^ tympanic membrane abnormalities,^9^, ^10^ neonatal^9^ and post-natal^32^ icterus, convulsions, and hospitalization.^9^ On the day of the evaluation, self-reported associated signs were also included, such as sinusitis, cold, earache and use of ventilation tube.^20^ Low socioeconomic level,^18^, ^32^ income,^8^, ^9^, ^15^ education level^12^, ^15^ and low maternal education level^28^ were associated with HI. Untreated middle ear infections, in the case of limited access to pediatric care, constitute an important risk factor for HI.

Variation in the prevalence among adolescents was verified herein, depending on the normality criterion utilized, as some studies analyzed incapacitating hearing loss,^11^, ^12^, ^15^, ^16^, ^18^ while others included frequencies over 4 kHz in the normal hearing criterion,^7^, ^8^, ^9^, ^17^, ^19^, ^24^, ^27^, ^30^ evidencing the importance of evaluating high frequencies in this group. The four studies that focused on adolescents as main investigated subjects were carried out within the last decade, and the prevalence found varied between 2.2%^30^ and 34.88%.^24^ The highest prevalence can be explained by the inclusion of frequencies over 8 kHz and evoked optoacoustic emissions. It is possible that this occurred due to noise exposure when using personal devices,^17^, ^23^ as the use of ear- and head-phones is common, without concerns regarding the exposure levels or duration.^6^

The use of media technologies must be highlighted, as well as the habit of listening to music with ear- and head-phones, which occurs progressively earlier in life,^34^ and therefore it is common to be precociously exposed to high levels of noise. A study involving school-aged individuals, aged between 6 and 14 years old in Poland, investigated lowered thresholds in high frequencies – 6–8 kHz, altered in 17.8% of the sample, being the influence of noise the most probable factor for such change.^7^ It is important to mention that the classifications for hearing loss generally do not include high frequencies, such as the classification proposed by the WHO and employed in some of the included studies.^9^, ^12^, ^15^ Some of the screenings carried out did not include high frequencies, and therefore might not have evidence the beginning of noise-induced hearing loss, which surely presents high incidence in this specific population, as revealed by the increase in HI prevalence in adolescents over a time interval of almost ten years.^8^ There was an association between the use of ear- and head- phones and academic issues,^9^ highlighting the importance of auditory health interventions.

Overall, it is difficult to compare the prevalences encountered in different studies,^19^ as demonstrated in the results presented herein. Besides the heterogeneity of the methods employed to detect and classify HI in school-aged children and adolescents, the life context and the health of this population is diverse, and so are the auditory changes experienced by younger and older children.^15^ These factors interfere with HI prevalence, constituting the main limitation of the study presented herein. Despite the heterogeneity of methods, prevalence and its associated factors, HI is an important factor that compromises the academic development and performance of children and adolescents.

## Conclusion

There is heterogeneity regarding methodology, normality criteria, and consequently, regarding prevalence and its associated factors. Nevertheless, the relevance of the subject and the necessity of early interventions are unanimous across studies. More studies are required, locally and globally, to investigate the correlation between the associated factors and hearing impairment in this population, so that auditory health interventions and public policies are progressively more assertive and directed to the new necessities of this generation.

## Conflicts of interest

The authors declare no conflicts of interest.
